# Pathogenesis and Targeted Therapy of Epilepsy

**DOI:** 10.3390/biomedicines10123134

**Published:** 2022-12-05

**Authors:** Prosper N’Gouemo

**Affiliations:** Department of Physiology and Biophysics, Howard University College of Medicine, Washington, DC 20059, USA; prosper.ngouemo@howard.edu; Tel.: +1-202-806-9708

The *Biomedicines* Special Issue (BSI) of “Pathogenesis and Targeted Therapy of Epilepsy” seeks papers providing new insights into the roles of voltage-gated and ligand-gated ion channels and their related signaling in the pathogenesis and pathophysiology of acquired epilepsy and inherited epilepsy. We are pleased that several renowned researchers have contributed to this first edition of BSI, comprising seven original articles and four reviews. Topics in this BSI include the identification of altered postsynaptic glutamate receptors as a potential mechanism underlying epileptogenesis in the hippocampus using the 4-aminopyridine in vitro model of epileptiform activity [1]. Another report suggests the activation of Ca^2+^-activated chloride channels as a novel cellular mechanism for suppressing acoustically evoked generalized tonic-clonic seizures in the strain of the genetically epilepsy-prone rats exhibiting moderated seizure severity [2]. Using another model of acoustically evoked seizures (Wistar Audiogenic Rat, WAR), Lazarini-Lopes et al. [3] report that TRPV1 channels might contribute to the cellular mechanism underlying epileptogenesis and anxiety-like behavior following repetitive episodes of seizures. Furthermore, a comprehensive review discusses the relationship between acoustically evoked seizure susceptibility and post-ictal catalepsy and motor hyperactivity [4]. In another token, Kim and Kang [5] provide evidence of an upregulation of tandem of the P domain in weak inwardly rectifying K^+^ channels (TWIK)-related acid-sensitive K^+^-1 (TASK-1) channels in hippocampal CA1 astrocytes in the pilocarpine post-status epilepticus model of temporal lobe epilepsy. Another report from the same group reveals that co-treatment with an elective TASK-1 inhibitor and levetiracetam (LEV) reduced the severity of LEV refractory seizures. Additional studies from Kim et al. [6] provide evidence that dysregulation of AKT/GSK3b/CREB-mediated glutamate ionotropic receptor AMPA type 1 subunit (GRIA1) surface expression may contribute to AMPA receptor antagonists’ refractory seizures in the pilocarpine model of temporal lobe epilepsy. Similarly, dysregulation of PP2B-ERK1/2-SGK1-NEDD4-2-mediated GRIA1 ubiquitination may also contribute to AMPA receptor antagonists’ refractory seizures [7]. In a model of traumatic brain injury (TBI), Wang et al. [6] report that peripheral infection after TBI increases neuronal excitability and facilitates post-traumatic epileptogenesis in the pentylenetetrazole model of seizures [8]. Furthermore, Ndoke-Ekane et al. [9] provide evidence that magnetic resonance imaging (MRI) improves the placement accuracy of intracerebral electrode implantation and that chronically implanted electrodes do not increase cortical and hippocampal atrophy in a rat model of post-traumatic epilepsy. A review by Yamanaka et al. [10] discusses the neuroinflammatory role of brain pericytes in epilepsy. Finally, another study by Bucknix et al. [11] examines the potential mechanisms underlying the anticonvulsant effects mediated by the orexigenic peptide ghrelin.

Epilepsies are disorders of neuronal excitability characterized by the occurrence of spontaneous, repeated episodes of seizures, and their incidence rate continues to increase yearly. Although many antiseizure medications (ASM) are available, about 30% of epileptic patients have ASM-refractory seizures. Thus, there is a need to develop new therapies to mitigate epileptogenesis and ASM-refractory seizures based on novel molecular targets for controlling neuronal hyperexcitability that leads to seizures. This first edition of BSI provides evidence of novel molecular targets for controlling epileptogenesis, generalized tonic-clonic seizures, and ASM-refractory seizures.
